# On the Cryptogamous Origin of Malarious and Epidemic Fevers

**Published:** 1849-06

**Authors:** 


*On the Cryptogamous origin of Malarious and Epidemic fevers.* By J. K. Mitchell, A. M., M. D. Prof. of practical medicine in the Jefferson Medical College of Philadelphia.
Philadelphia: Lea & Blanchard, 1849, 12 mo, pp 137.

It is seldom that one enters on the reading of a book without some prejudgment of its contents. Our prepossessions are based upon our knowledge of the author, or his previous productions; upon the subject, or scope of the work; its general appearance, and various other circumstances. We are disposed to like, or dislike a book, as we are an individual, before familiar acquaintance, by the first impressions we receive from his physiognomy, or by what we have heard of him or his actions. Hence our too hastily formed notions often prove incorrect, and require to be modified or abandoned. Our own experience with reference to this volume has suggested these reflections. Our anticipations on reading the title page, were quite different from the sentiments left by a careful perusal of the work. We could not at first avoid feeling regret that the author had identified himself with a fanciful hypothesis, which would only serve as a fresh occasion for sarcasm for those who search the annals of medical literature for subjects of ridicule or reproach. Right glad are we that we did not follow the ad-advice attributed to the late Rev. Sidney Smith, and review the work without reading it, in order to avoid the prejudicies which an intimate acquaintance with an author's production is apt to engender!

By this strain of remark we would not be understood to imply that, in our opinion, the cryptogamous origin of malarious and epidemic diseases is satisfactorily established, or, even rendered, in any degree probable. The theory appears not less fanciful *after* than *before* perusal of the work. But the apprehensions of the reader lest the philosophical character of the author may be compromised, is relieved, by ascertaining that he does not claim to have succeeded in establishing the curious doctrine which he advocates. He proposes only, to use his own language, to have made an excursion into a new field of "mingled reason and fancy." The ability displayed in the discussion, the many facts collected, and the interesting considerations adduced, render the work attractive and valuable, irrespective of the cryptogamous theory. We may go further than this, and say that, although the ingenuity of the author will probably fail in making many converts to his theory, be it true or false, the careful reader will be satisfied that the author has rendered valuable service to science by clearly defining the precise state of our knowledge, or rather of our ignorance of the Etiology of miasmatic and epidemic diseases. If he has not discovered the true *semina morborum* in the invisible spores of fungi, (which we presume he has not,) he has shown that other theories which have been, are still more or less in vogue, are untenable—the hypothesis of marsh miasma not excepted. In so far, the author has accomplished something for science, since to know how little we do know is a pre-requisite for the advancement of knowledge. Regarded in comparison with other theories, the claims of the fungous theory are by no means contemptible. It may safely be said that the latter is sustained by as plausible arguments as any other, but this, unfortunately, is damning with faint praise.

Under these circumstances we have no right to quarrel with any one for a theoretical excursion into a field as tempting to fancy and reason, as it has proved unfruitful to those who have hitherto essayed to explore it. With these desultory comments we commend the work to our readers, promising them an agreeable occupation for the few hours that its perusal will require. The size of the work, as will be perceived, is small. It should be added, that it consists of six lectures, delivered at the institution with which the author is connected, and is published in compliance with the solicitations of those who listened to their recital.

That our readers may form some idea of the theory, and the arguments upon which it rests, we subjoin a brief recapitulation, in which the author gives a summary of the points which it is his object, in the several lectures, to establish, or illustrate.—

I have now, gentlemen, brought to a close the prolonged examination of the cause of miasmatic fevers, and non-contagious epidemics. Let me recapitulate, in a very cursory way, the most important elements of our argument.

I began, by showing that all the usually received opinions on this subject, are liable to insuperable objections, except that which refers to the causation by organic life, and especially by animalcules, as held by Columella, Kircher, Linnæus, Mojon, and Henry Holland.

While I was impressed, for the reasons so ably stated by Holland, with the greater probability of the organic theory, I prefer, for reasons stated by myself, the fungous, to the animalcular hypothesis.

My preference is founded on the vast number, extraordinary variety, minuteness, diffusion and climatic peculiarities of the fungi.

The spores of these plants are not only numerous, minute, and indefinitely diffused, but they are so like to animal cells, as to have the power of penetrating into, and germinating upon the most interior tissues of the human body.

Introduced into the body through the stomach, or by the skin or lungs, cryptogamous poisons were shown to produce diseases of a febrile character, intermittent, remittent and continued; which were most successfully treated by wine and bark.

Many cutaneous diseases, such as *favus* and *mentagra*, are proved to be dependent upon cryptogamous vegetations; and even the disease of the mucous membrane, termed aphthæ, arises from the presence of minute fungi.

As microscopic investigations become more minute, we discover protophytes in diseases, where, until our own time, their existence was not even suspected, as in the discharges of some kinds of dysentery, and in the *sarcina* of pyrosis. We are therefore entitled to believe that discovery will be, on this subject, progressive.

The detection of the origin of the muscardine of the silk worm, and a great many analogous diseases of insects, fishes and reptiles, and the demonstration of the cryptogamism of these maladies, their contagious character in one species of animals, their transfer to many other species, nay, even to vegetables themselves, all concur to render less improbable, the agency of fungi in the causation of diseases of a febrile character.

A curious citation was subsequently made, of the fungiferous condition during epidemies and epizootics. These moulds, red, white, yellow, gray, or even black, stained garments, utensils and pavements, made the fogs fetid, and caused disagreeable odors and spots, even in the recesses of closets and the interior of trunks and desks.

These moulds existed, even when the hygrometric state did not give to the air any unusual moisture for their sustentation and propagation. Their germs seem to have, as have epidemics, an inherent power of extension.

The singular prevalence of malarious diseases in the autumn, is best explained by supposing them to be produced by the fungi, which grow most commonly at the same season. The season of the greatest photophytic activity, is, in every country, the period of the greatest malarious disturbance. The sickly season is, in the rains of Africa, in the very dry season in Majorca and Sardinia, in the rainy season of the insular West Indies, and in the dry season of Demorara and Surinam. Even when the vegetation is peculiarly controlled, as in Egypt by the Nile, and the cryptogami are thus thrown into the season of winter and spring, that season becomes, contrary to rule, the pestilential part of the year.

Marshes are a safe residence by day, whilst they are often highly dangerous by night. In the most deadly localities of our southern country, and of Africa, the sportsman may tread the mazes of a swamp safely by day, although at every step, he extricates vast quantities of the gases which lie entangled in mud and vegetable mould. This point, so readily explained by reference to the acknowledged nocturnal growth and power of the fungi, is a complete stumbling-block to the miasmatists.

The cryptogamous theory well explains the obstruction to the progress of malaria offered by a road, a wall, a screen of trees, a veil or a gauze curtain.

It also accounts for the nice localization of an ague, or yellow fever, or cholera, and the want of power in steady winds to convey malarious diseases into the heart of a city, from the adjacent country.

It explains also, well, the security afforded by artificially drying the air of malarious places, the exemption of cooks and smiths from the sweating sickness, the cause of the danger from mouldy sheets, and of the sternutation from old books and papers.

On no other theory can we so well account, if account at all, for the phenomena of milzbrand and milk-sickness, the introduction of yellow fever into northern ports, and the wonderful irregularities of the progress of cholera.

The cryptogamous theory will well explain the peculiar domestication of different diseases in different regions, which have a similar climate; the plague of Egypt, the yellow fever of the Antilles, and the cholera of India. It accounts, too, for their occasional expansion into unaccustomed places, and their retreat back to their original haunts.

Our hypothesis will also enable us to tell, why malarious sickness is disproportionate to the character of the seasons; why it infests some tropical countries and spares others; why the dry Maremma abounds with fevers, while the wet shores of Brazil and Australia actually luxuriate in healthfulness. The prolonged incumbative period, the frequent relapses of intermittents, and the latency of the malarious poisons for months, can only be explained by adopting the theory of a fungous causation.

Finally, it explains the cause of the non-recurrence of very potent maladies, better than the chemical theoroy of Liebig; and shows why the earliest cases of an epidemic are commonly the most fatal.

When I entered upon the task of elucidating for you this very difficult subject, gentlemen, I did not dream of its extent and importance, nor did I suppose that it would have imposed upon me so much research, or inflicted upon you so many lectues.

I have, therefore, not attempted to account by this theory, for the periodicity of malarious diseases, rather for want of time than want of power, and from a desire not to tax too severely your patience.

